# Supplementary material for: Color-tunable single-fluorophore supramolecular system with assembly-encoded emission
Source: Nat Commun. 2020 Jan 9;11:158. doi: 10.1038/s41467-019-13994-6 (PMC6952351; doi:10.1038/s41467-019-13994-6)
Supplement: Supplementary file 1 — Supplementary Information [file 41467_2019_13994_MOESM1_ESM.pdf]

Supplementary Information

Color-Tunable Single-Fluorophore Supramolecular System with Assembly-Encoded Emission

Wang et al.

Compounds I, II, III were synthesized according to previous reports[1-3].

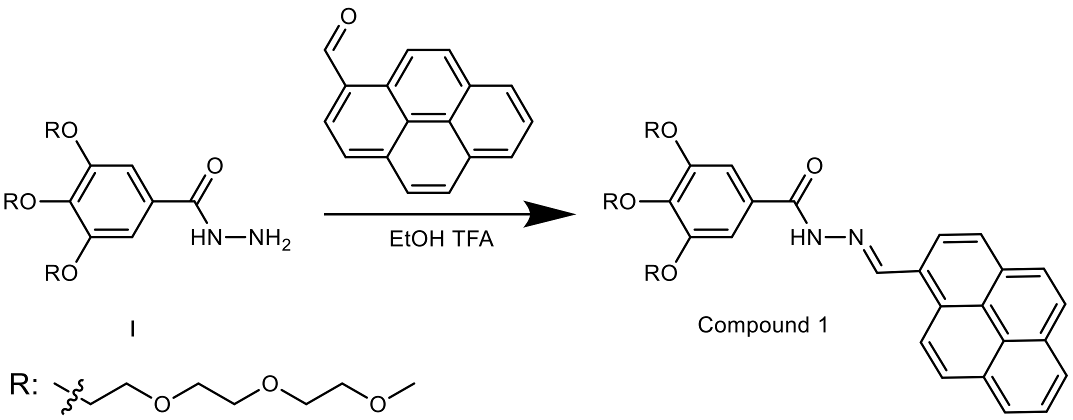

Supplementary Figure 1. Synthesis of compound 1.

I (0.2 g, 0.32 mmol), 1-Pyrenecarboxaldehyde (0.074 g, 0.32 mmol), trifluoroacetic acid (10 μL) was mixed with ethanol (40 mL) and refluxed for 24 hours under stirring in an argon gas atmosphere. Then the reaction mixture was cooling down to room temperature. After evaporation of the solvent, the residue was purified by silica gel column chromatography (CH_2Cl_2/MeOH = 15: 1 as eluent) to give compound 1 (0.694 g, 47%) as bright yellow oil. Yield: 0.22 g (81%).^{1}H NMR ($CDCl_3$, 500 MHz, 298 K): σ 11.12 (s, 1H), 9.58 (s, 1H), 8.72 (d, *J* = 7.5 Hz, 1H), 8.64 (d, *J* = 9.5 Hz, 1H), 8.18 (d, *J* = 7.5 Hz, 1H), 8.14 (t, *J* = 5.0 Hz, 2H), 8.07 (d, *J* = 8.5 Hz, 2H), 8.01 (d, *J* = 9.0 Hz, 1H), 7.98 (t, *J* = 7.5 Hz, 1H), 7.40 (s, 2H), 4.22 - 4.18 (m, 4H), 4.17 - 4.11 (m, 2H), 3.81 - 3.76 (m, 4H), 3.76 - 3.72 (m, 2H), 3.56 - 3.52 (m, 6H), 3.36 (s, 3H), 3.31 (s, 6H). ^{13}C NMR ($CDCl_3$, 101 MHz, 298 K): σ 164.37, 152.45, 147.72, 141.60, 132.41, 131.02, 130.27, 129.41, 128.31, 128.12, 127.27, 126.77, 125.91, 125.69, 125.34, 125.06, 124.82, 124.47, 124.28, 121.87, 107.65, 77.51, 77.19, 76.87, 72.36, 71.83, 70.60, 70.43, 69.58, 68.78, 58.97, 58.88. HRMS (ESI) (m/z): $[M + Na]^+$ calcd for $C_{45}H_{58}N_2O_{13}Na^+$, 857.3831; found, 857.3839.

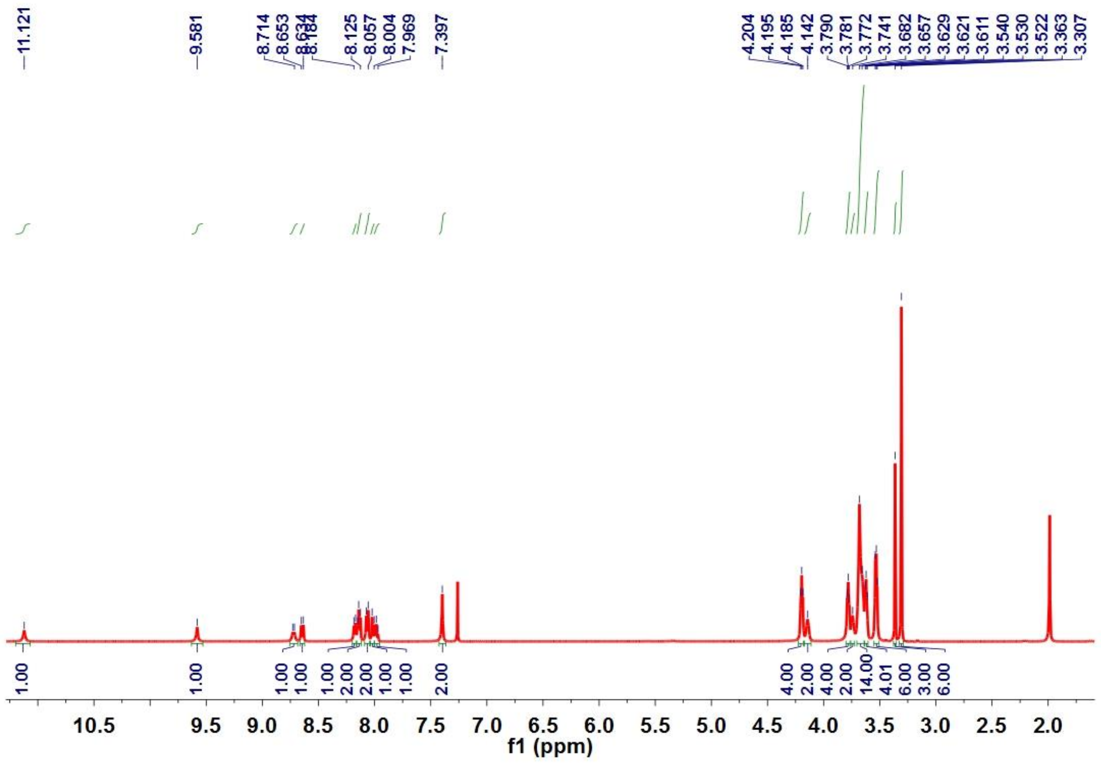

Supplementary Figure 2. ^{1}H NMR spectrum of compound 1.

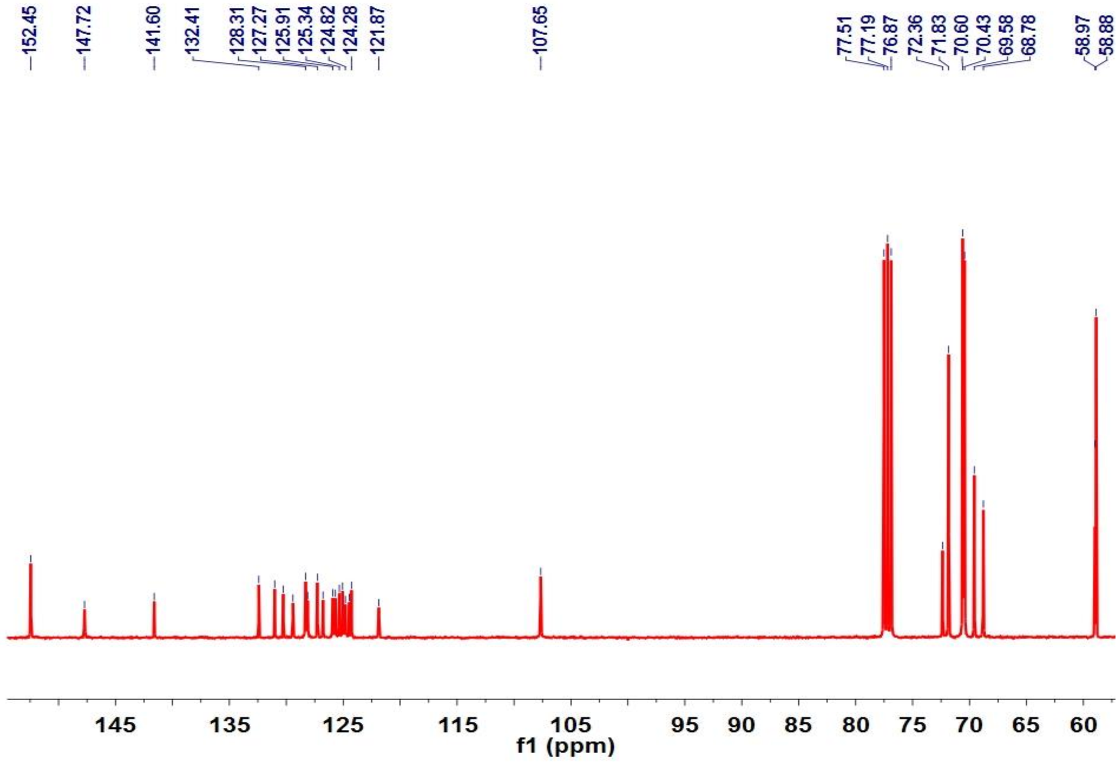

Supplementary Figure 3. ^{13}C NMR spectrum of compound 1.

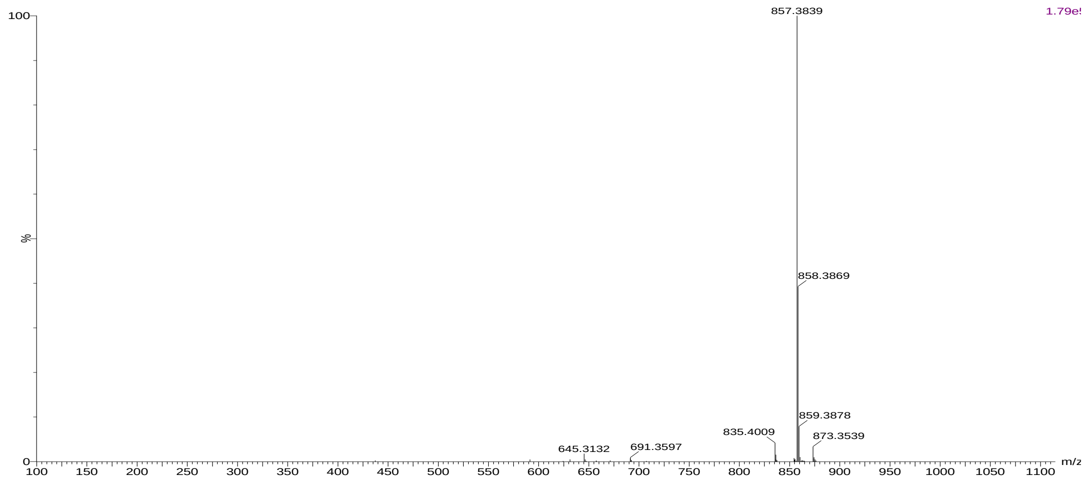

Supplementary Figure 4. Mass spectrum of compound 1.

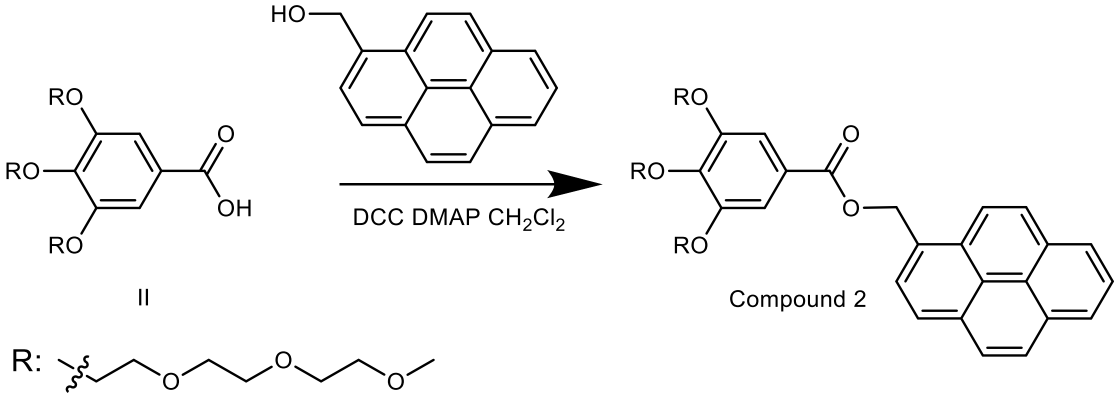

Supplementary Figure 5. Synthesis of compound 2.

Compound 2 was synthesized by the esterification reaction between 1-Pyrenemethanol and 2. A mixed solution of II (0.2 g, 0.328 mmol), 1-Pyrenecarboxaldehyde (0.076 g, 0.328 mmol), 4-dimethylaminopyridine (DMAP) (2 mg) in 30 mL dichloromethane (CH_2Cl_2), dicyclohexylcarbodiimide (DCC) (0.068 g, 0.33 mmol) in 10 mL dichloromethane was added dropwise at 273 K. The mixture solution was stirred at room temperature for 18 hours. The mixture was filtered to remove the precipitation and the filtrate was evaporated under reduced pressure. The residue was purified by column chromatography (SiO_2, CH_2Cl_2/MeOH = 30: 1 as eluent) to get the product compound 2 as brown oil (0.21 g, 76%).^{1}H NMR ($CDCl_3$, 500 MHz, 298 K): σ 8.36 (d, *J* = 9.0 Hz, 1H), 8.25 - 8.15 (m, 4H), 8.12 (d, *J* = 8 Hz, 1H), 8.09 (d, *J* = 9.5 Hz, 1H), 8.07 (d, *J* = 9.0 Hz, 1H), 8.03 (t, *J* = 7.5 Hz, 1H), 7.31 (s, 2H), 6.04 (s, 2H), 4.20 (t, *J* = 5.0 Hz, 2H), 4.12 (t, *J* = 4.9 Hz, 4H), 3.80 (t, *J* = 5.0 Hz, 4H), 3.77 (t, *J* = 5.0 Hz, 2H), 3.70 - 3.63 (m, 6H), 3.63 - 3.53 (m, 12H), 3.52 - 3.46 (m, 6H), 3.36 - 3.32 (m, 9H). ^{13}C NMR ($CDCl_3$, 101 MHz, 298 K): σ 166.12, 152.31, 142.80, 131.81, 131.22, 130.72, 129.72, 128.91, 128.27, 127.86, 127.38, 126.11, 125.52, 124.93, 124.67, 122.97, 109.30, 77.37 77.05, 76.73, 72.39, 71.90, 70.60, 69.56, 68.86, 65.46,

58.98. HRMS (ESI) (m/z): [M + Na]$^+$ calcd for $C_{45}H_{58}O_{14}Na^+$, 845.3719; found, 845.3725.

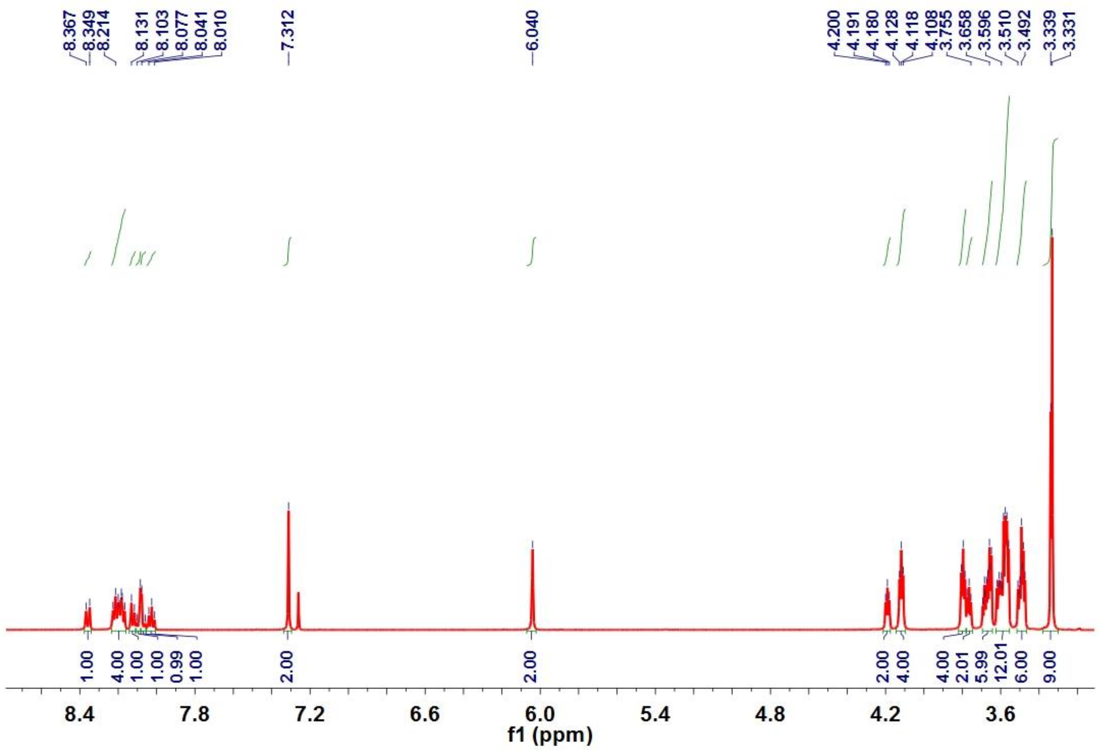

Supplementary Figure 6. ^{1}H NMR spectrum of compound 2.

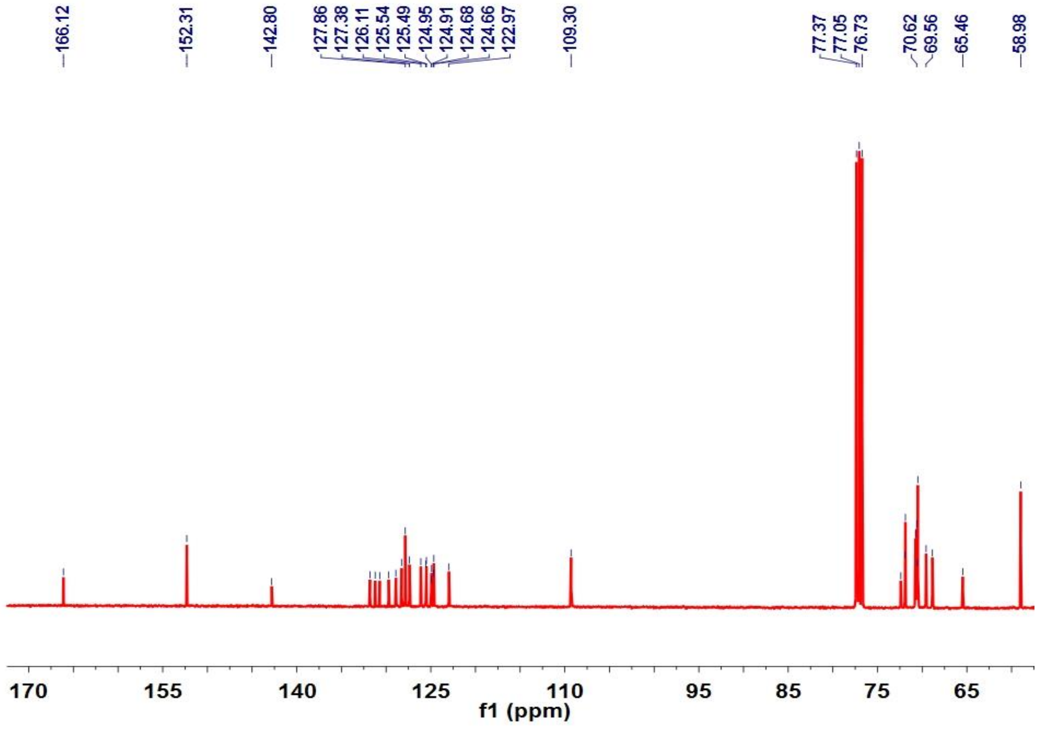

Supplementary Figure 7. ^{13}C NMR spectrum of compound 2.

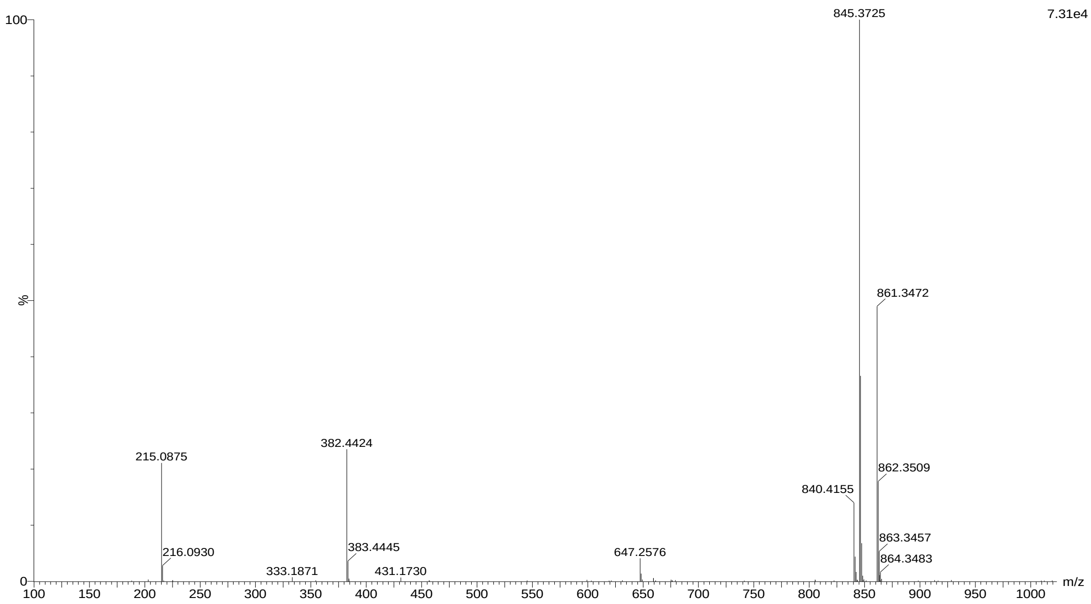

Supplementary Figure 8. Mass spectrum of compound 2.

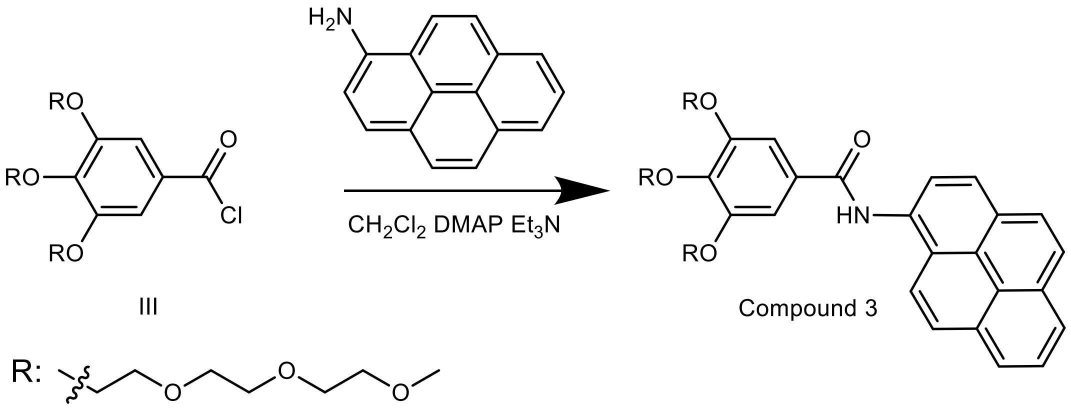

Supplementary Figure 9. Synthesis of compound 3.

III (0.514 g, 0.82 mmol) and 1-Pyrenamine (0.178 g, 0.82 mmol) were dissolved in 30 mL CH_2Cl_2, then Et_3N and 4-dimethylaminopyridine (DMAP) (2 mg) in 5 mL CH_2Cl_2 were added dropwise under stirring. The mixture solution was refluxed for 24 hours. The solvent was removed by evaporation and the residue was purified by column chromatography (SiO_2, CH_2Cl_2/MeOH = 25: 1 as eluent) to get the product compound 3 as brown oil (0.21 g, 53%).^{1}H NMR ($CDCl_3$, 400 MHz, 298 K): σ 9.94 (s, 1H), 8.29 - 8.21 (m, 5H), 8.13 - 8.11 (m, 3H), 8.01 (t, *J* = 8.0 Hz, 1H), 7.54 (s, 2H), 4.27 - 4.21 (m, 6H), 3.85 - 3.83 (m, 4H), 3.79 - 3.77 (m, 2H), 3.67 - 3.64 (m, 6H), 3.58 - 3.52 (m, 12H), 3.45 - 3.40 (m, 6H), 3.25 (s, 3H), 3.21 (s, 6H). ^{13}C NMR (MeOD, 101 MHz, 298 K): σ 168.93, 153.77, 142.55, 132.52, 132.34, 132.18, 131.13, 130.57, 128.67, 128.34, 127.43, 127.36, 126.57, 126.36, 126.20, 126.02, 125.91, 125.61, 123.51, 108.33, 73.67, 73.01, 72.95, 71.77, 71.70, 71.59, 71.55, 71.41, 71.36, 70.75, 70.00, 59.22,

59.18, 49.81, 49.59, 49.38, 49.17, 48.95, 48.74, 48.53. HRMS (ESI) (m/z): $[M + Na]^+$ calcd for $C_{44}H_{57}NO_{13}Na^+$, 830.3722; found, 830.3726.

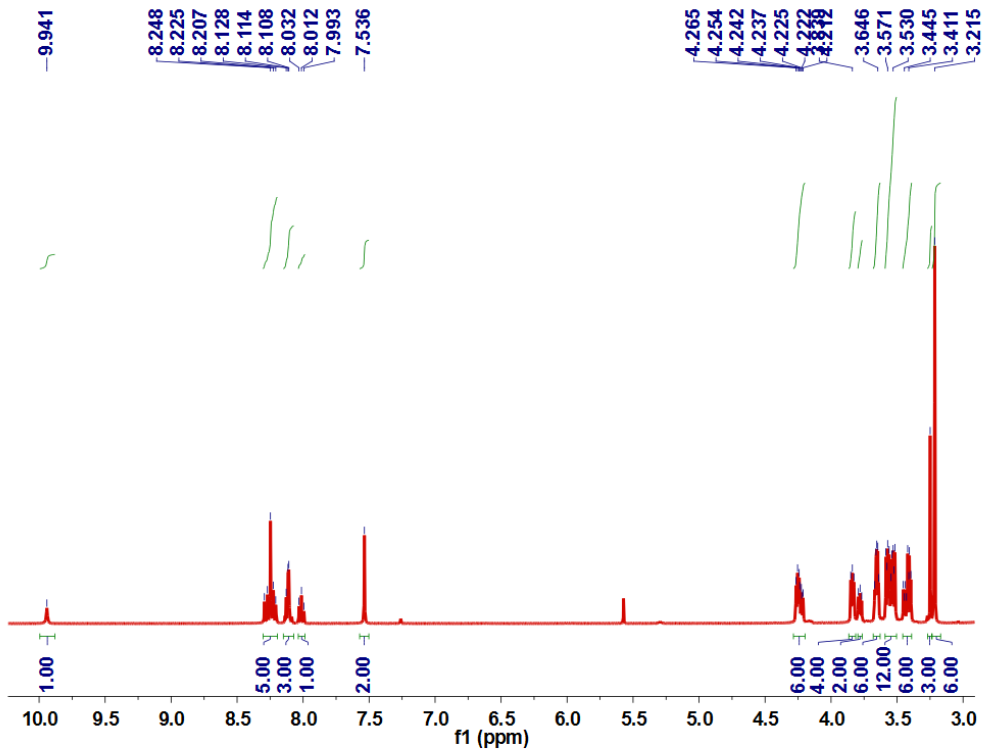

Supplementary Figure 10. ^{1}H NMR spectrum of compound 3.

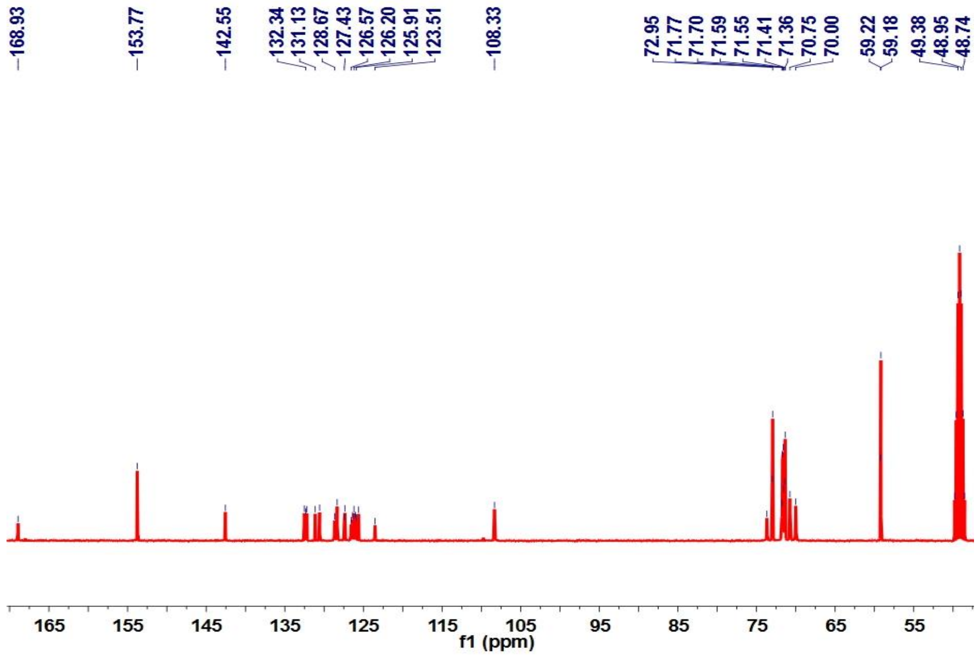

Supplementary Figure 11. ^{13}C NMR spectrum of compound 3.

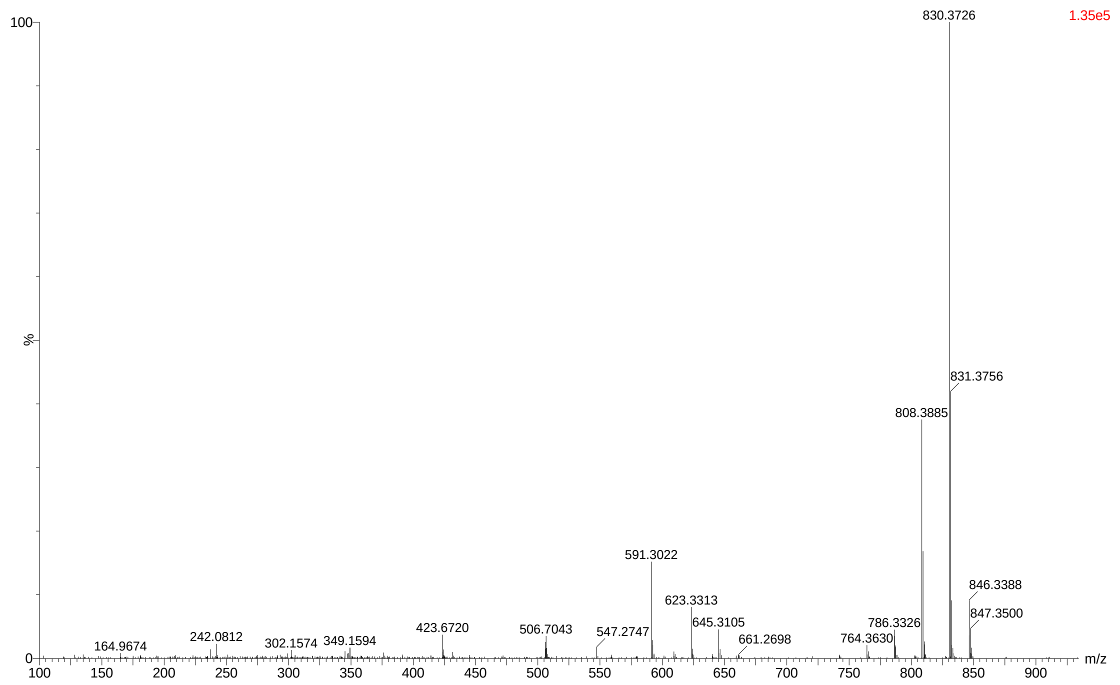

Supplementary Figure 12. Mass spectrum of compound 3.

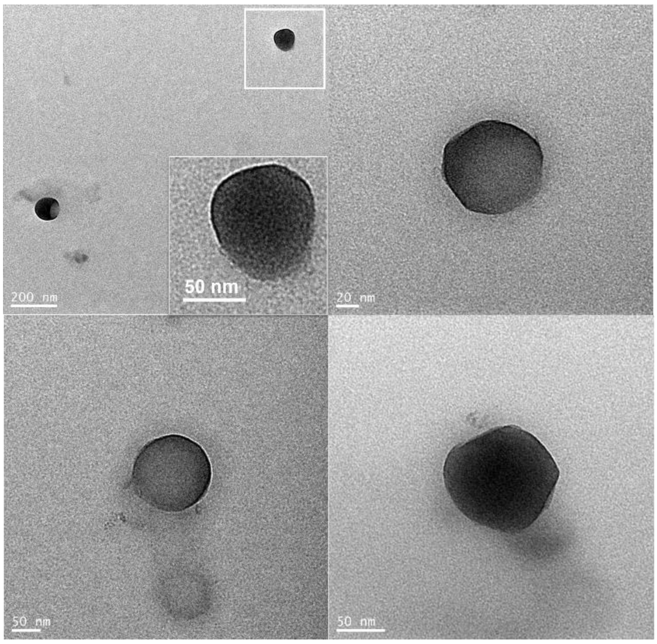

Supplementary Figure 13. TEM images of compound 1 in an aqueous solution (0.1 mM).

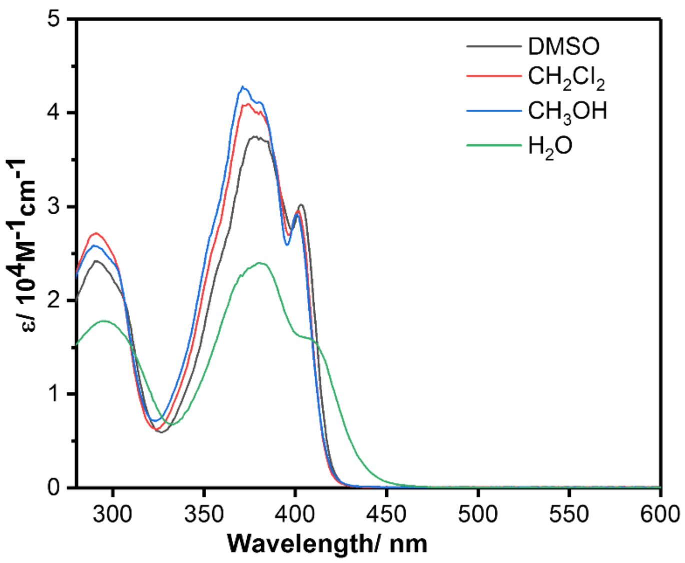

Supplementary Figure 14. UV-vis spectra of compound 1 in different solvents (30 μM).

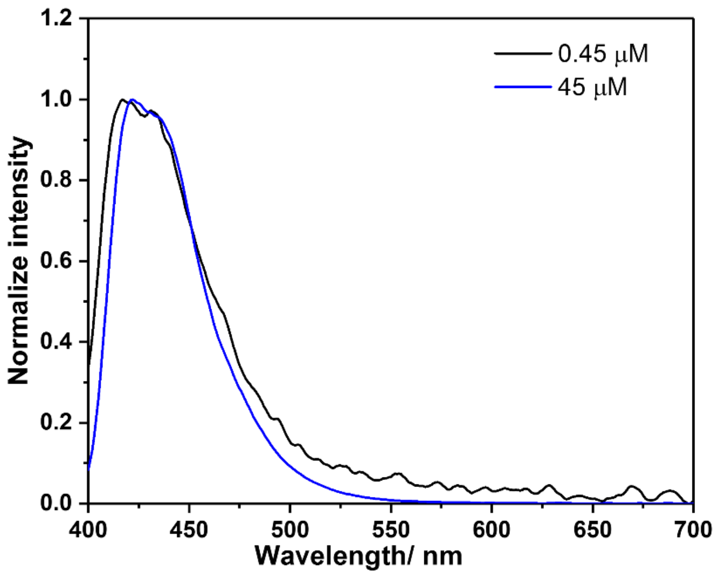

Supplementary Figure 15. Fluorescence spectra of compound 1 in CH_2Cl_2 with different concentrations, λ_{ex} = 365 nm.

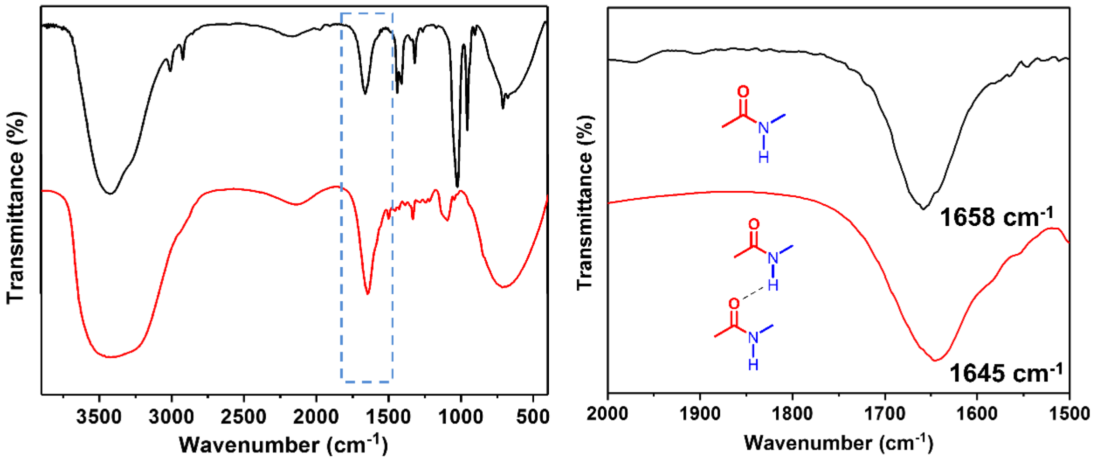

Supplementary Figure 16. FTIR spectra of compound 1 (10 mM) in DMSO (black line) and water (red line). The sharp amide I band shifted from 1658 cm^{-1} in DMSO (monomers) to 1645 cm^{-1} in H_2O (aggregation), suggesting the formation of intermolecular hydrogen bonds.

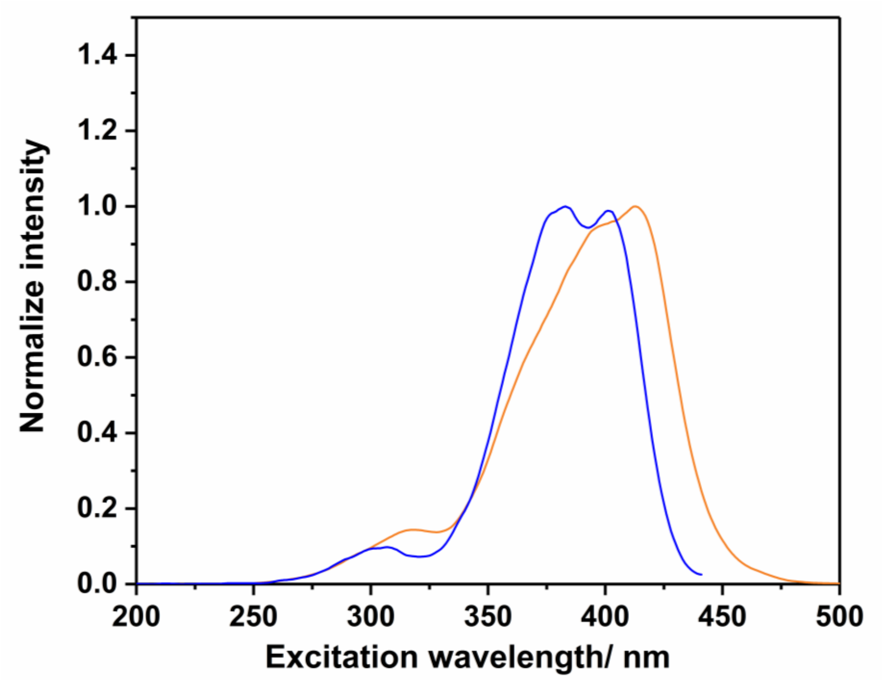

Supplementary Figure 17. Excitation spectra of compound 1 in aqueous solution with different concentrations (blue line: 5 μM, λ_{em} = 460 nm; orange line: 20 μM, λ_{em} = 530 nm).

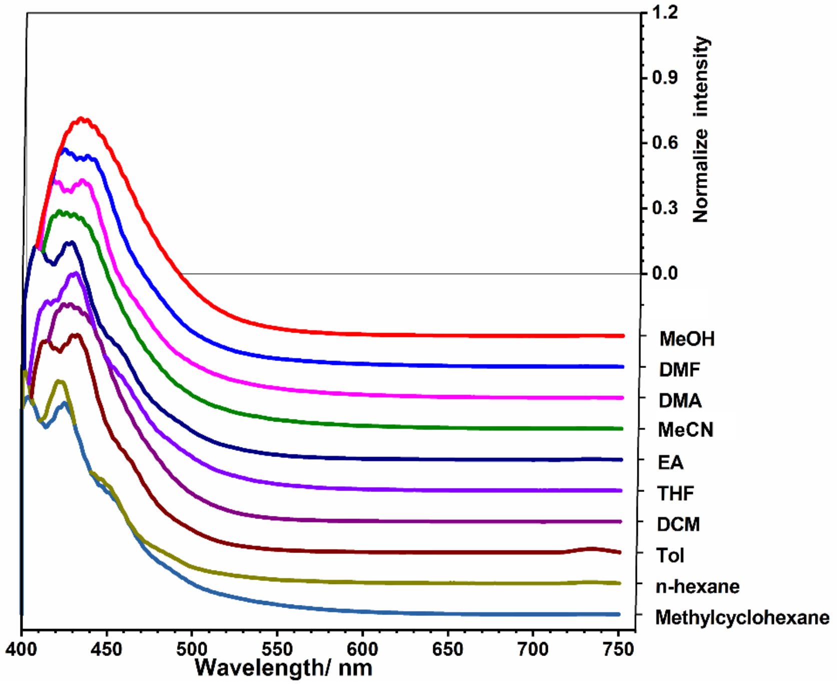

Supplementary Figure 18. Fluorescence spectra of compound 1 in different solvents (30 μM, λ_{ex} = 365 nm).

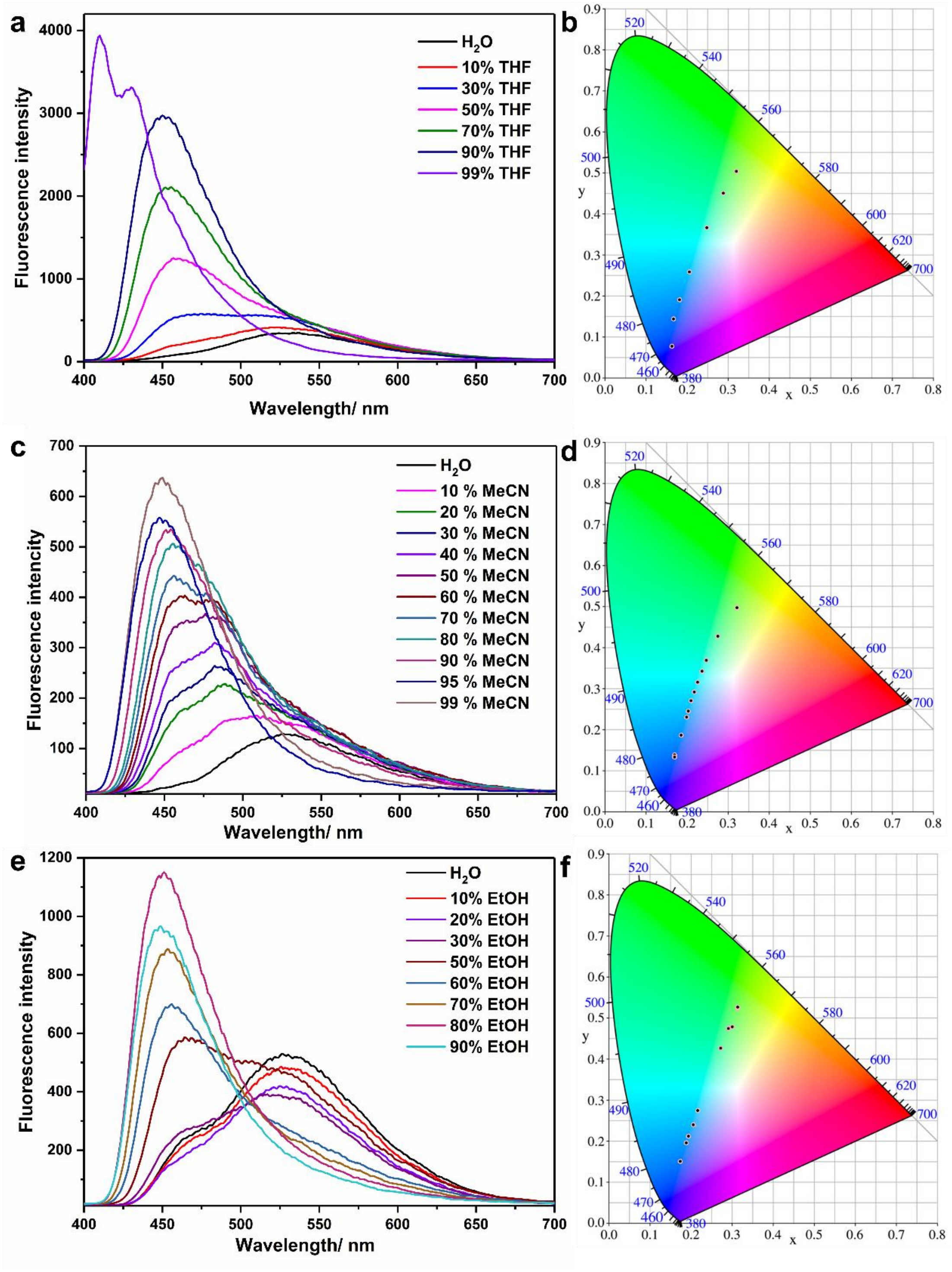

Supplementary Figure 19. Fluorescence spectra (a, c, e) and the chromaticity coordinates (b, d, f) of compound 1 in mixed solvents with different water fractions f_w (THF: 30 μM, MeCN: 30 μM, EtOH: 30 μM, λ_{ex} = 365 nm).

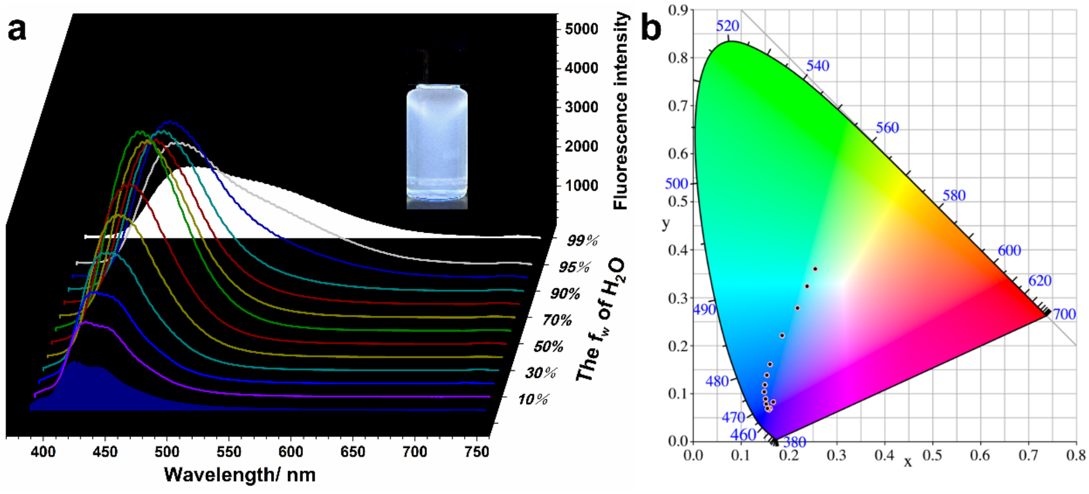

Supplementary Figure 20. Fluorescence spectra (a) and the chromaticity coordinates (b) of compound 1 in DMSO/H_2O mixtures with different f_w (20 μM, λ_{ex} = 365 nm). Inset: fluorescent images in DMSO/H_2O mixtures with f_w of 97%.

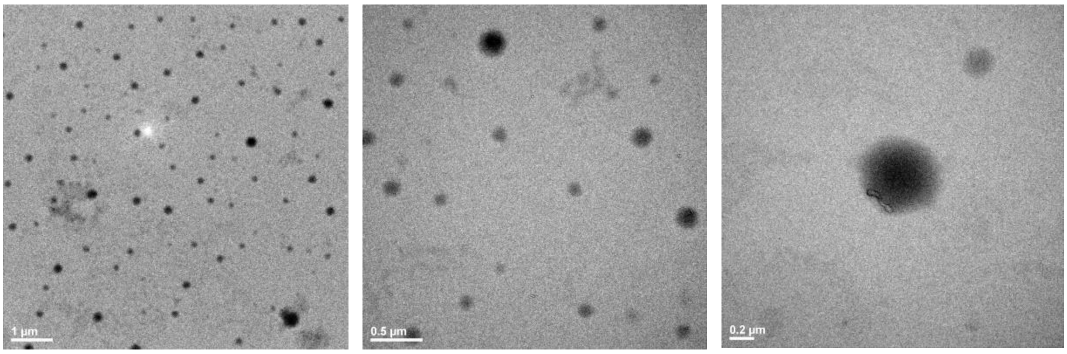

Supplementary Figure 21. TEM images of compound 2 in aqueous solution (0.1 mM).

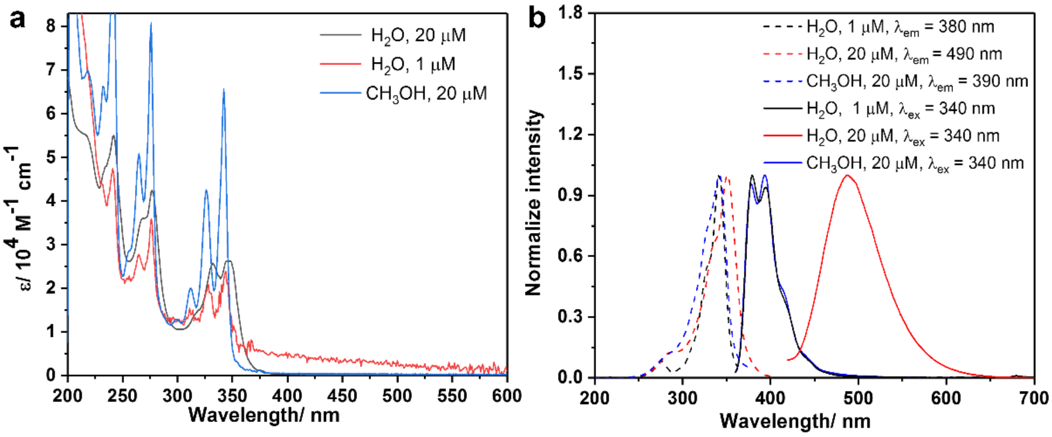

Supplementary Figure 22. UV-vis (a) and fluorescence spectra (b: solid line), excitation spectra (b: dash line) of compound 2 in MeOH and H_2O with different concentrations.

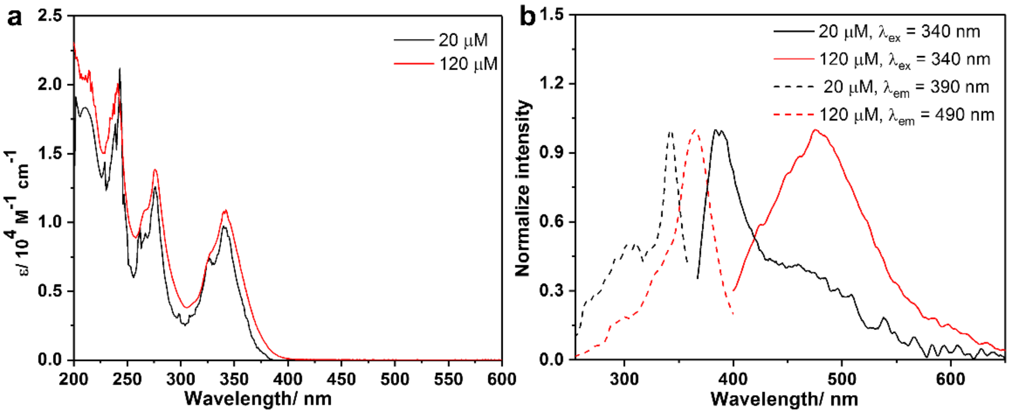

Supplementary Figure 23. UV-vis (a) and fluorescence spectra (b: solid line), excitation spectra (b: dash line) of compound 3. The broad absorption bands at 270 nm and 325 nm and the emission peak at 490 nm were observed in concentrated solution, suggesting the aggregation of compound 3.

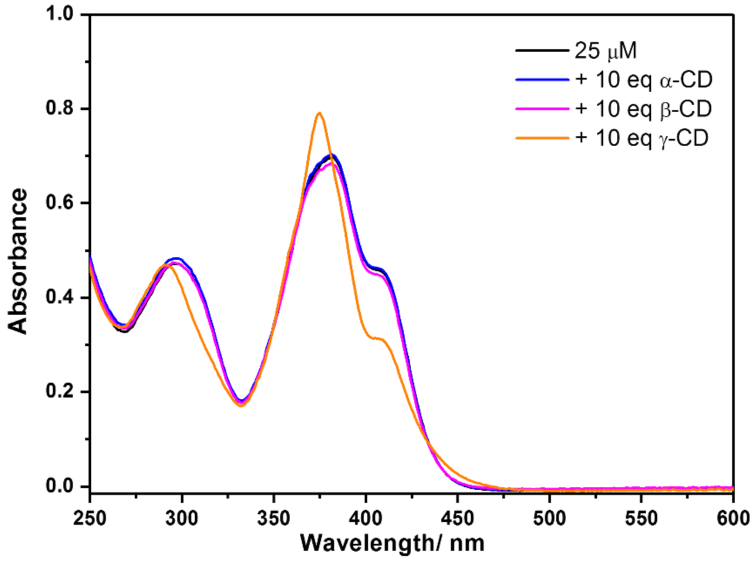

Supplementary Figure 24. The UV-vis spectra of compound 1 aqueous solution before and after the addition of α, β, γ-cyclodextrins.

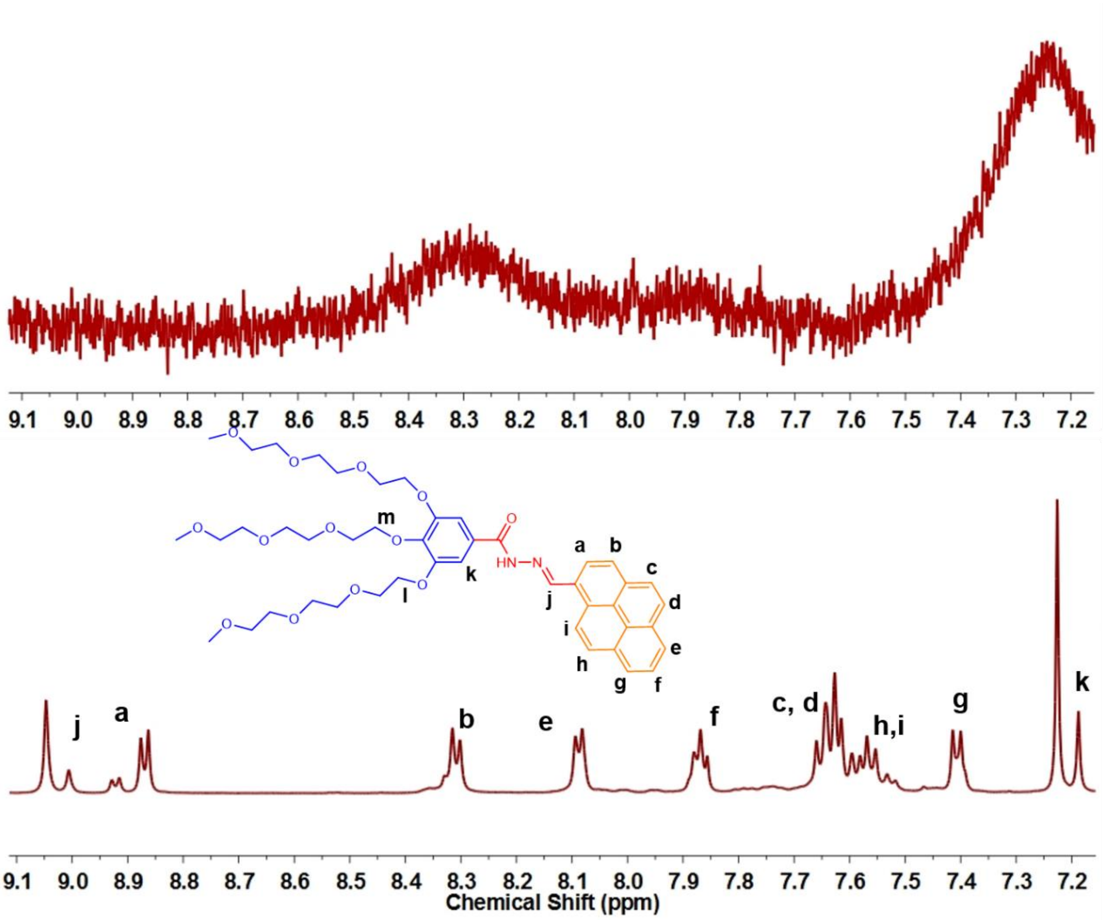

Supplementary Figure 25. ^{1}H NMR (D_2O) spectra of compound 1 (20 mM) prior (top) and after (bottom) addition of 2 eq γ-CD. In the aqueous solutions, the proton signals of pyrene were shielded owing to the formation of the vesicles. After adding γ-CD (2 eq) to compound 1 aqueous solution (20 mM), the successful disassembly of vesicles and subsequent inclusion of pyrene units in γ-CD can be suggested by following observations: i) the appearance of aromatic proton signals suggests the decrease of shielding effect; ii) proton signals H_a, H_j and H_k split into two groups.

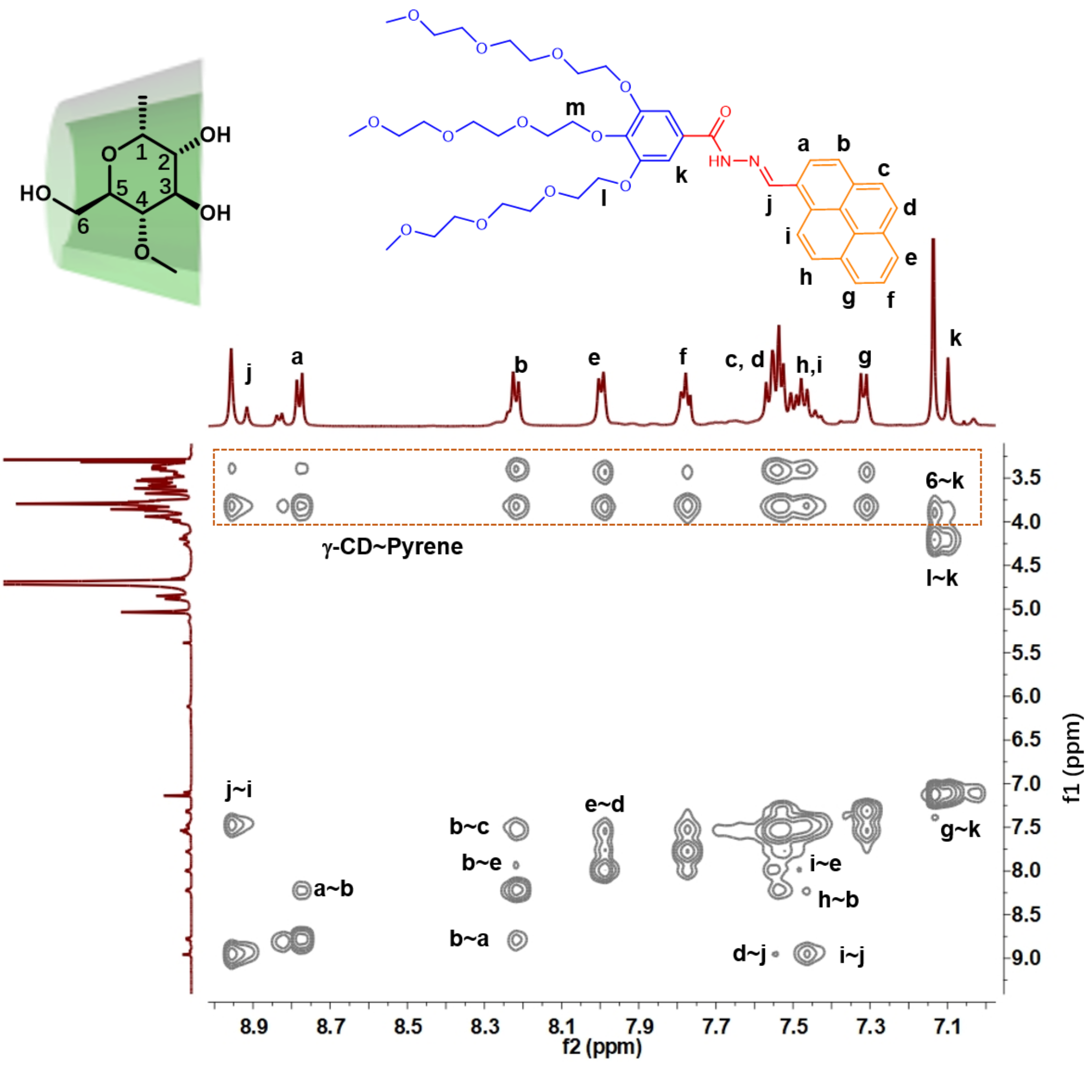

Supplementary Figure 26. 2D NOESY NMR spectrum of the complex (D_2O, 25°C). The correlation signals of γ-CD with pyrene and pyrene with pyrene were observed. Notably, the proton H_6 from the narrow rim of γ-CD was positioned close to proton H_k, suggesting that γ-CD is unidirectionally threaded onto the hydrophobic unit[4]. In addition, the dimer of pyrene was confirmed by the correlation of the signals of (b, e), (i, e), (h, b), (d, j) and (g, k).

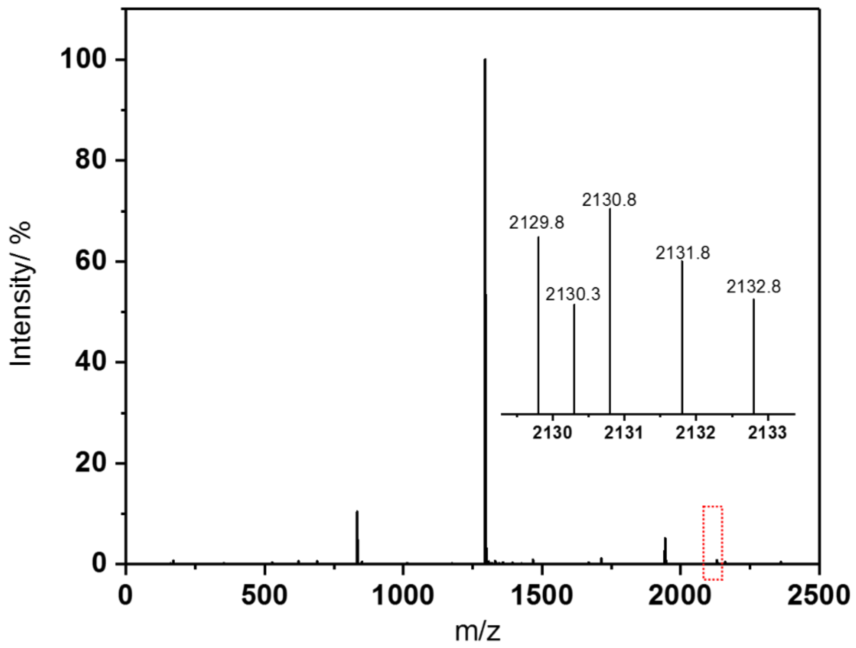

Supplementary Figure 27. Mass spectrum of compound 1 aqueous solution with 2 eq γ-CD. Weak signals of the quaternary complexes were found at 2130.8, which consistent with the result: [2M@2CD - 2H$^+$]$^{2-}$ calcd for $C_{186}H_{274}N_4O_{106}^{2-}$, 2130.8126 or [M@CD - H$^+$]$^-$ calcd for $C_{93}H_{137}N_2O_{53}^-$, 2130.8126. Furthermore, the difference of m/z between isotope signals 2129.8, 2130.3 and 2130.8 were 0.5, indicated the presence of two charge states, i.e. [2M@2CD - 2H$^+$]$^{2-}$. This result strongly confirmed that compound 1 and γ-CD self-assembled into 2:2 complex in water.

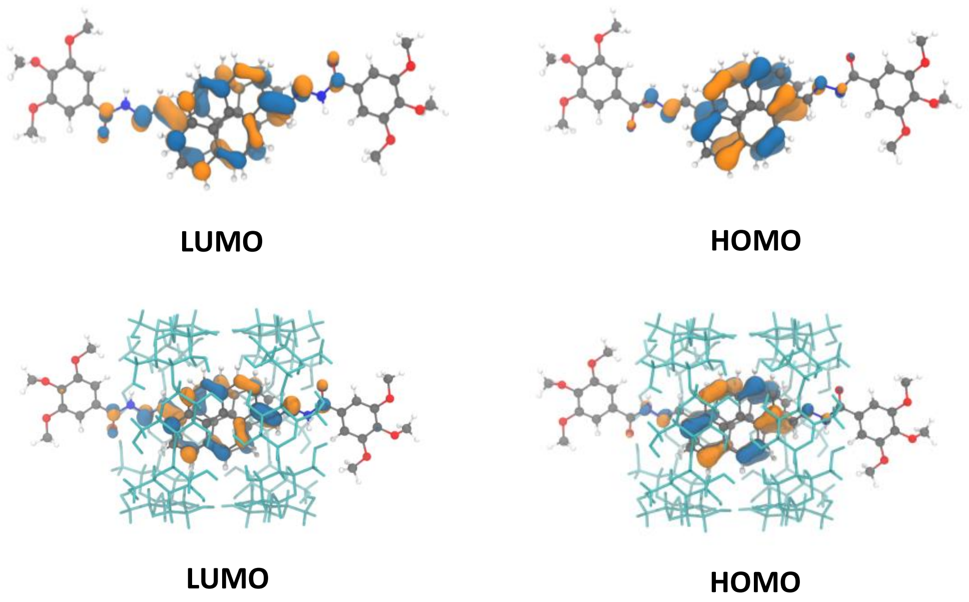

Supplementary Figure 28. The calculated geometries (top view) of the dimer and complex.

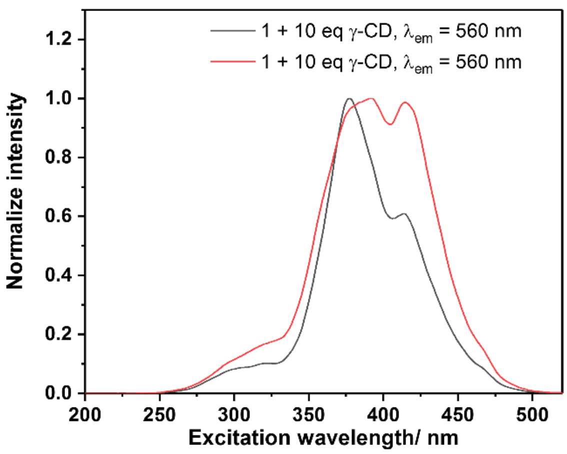

Supplementary Figure 29. Excitation spectra of compound 1 (black line: 5 μM, red line: 20 μM) in aqueous solution after the addition of γ-CDs.

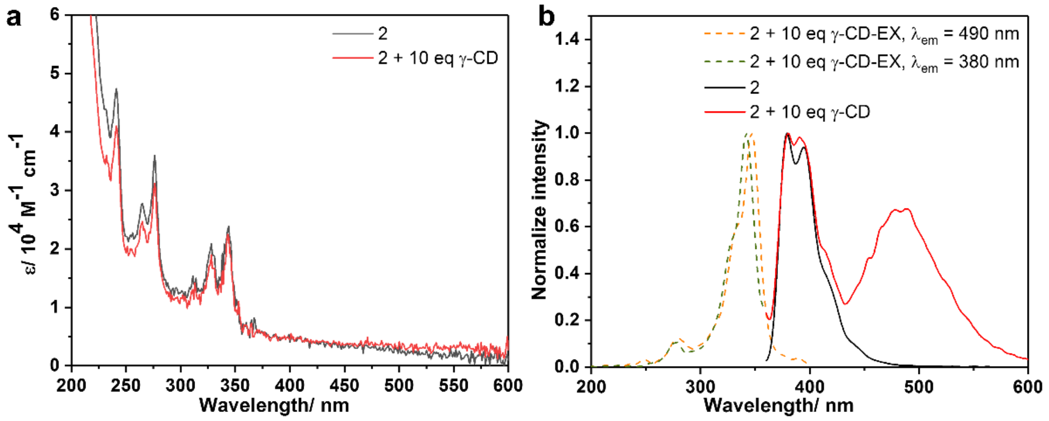

Supplementary Figure 30. UV-Vis (a) and fluorescence spectra (b: solid line), excitation spectra (b: dash line) of compound 2 (1 μM in H_2O) upon the addition of γ-cyclodextrins, λ_{ex} = 340 nm.

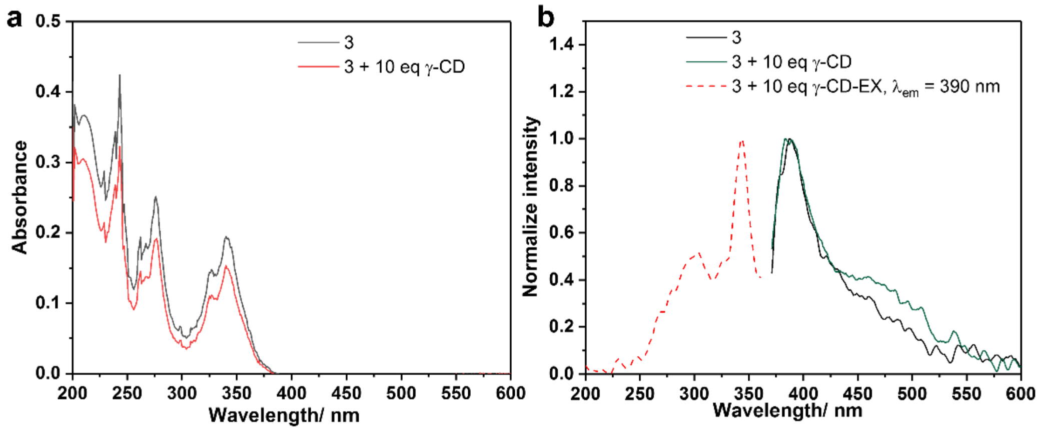

Supplementary Figure 31. UV-Vis (a) and fluorescence spectra (b: solid line), excitation spectra (b: dash line) of compound 3 (20 μM in H_2O) upon the addition of γ-cyclodextrins, λ_{ex} = 340 nm.

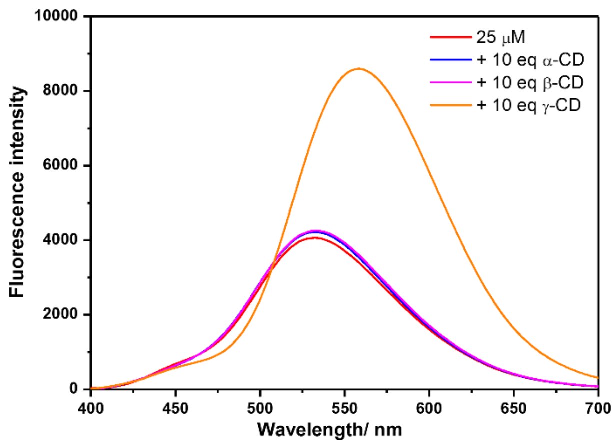

Supplementary Figure 32. The fluorescence spectra of compound 1 aqueous solution before and after the addition of α, β, γ-cyclodextrins (λ_{ex} = 365 nm).

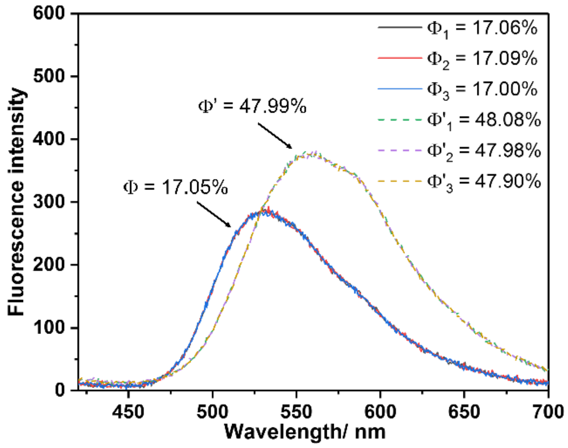

Supplementary Figure 33. The absolute solid fluorescence quantum yield of compound 1 (solid line) and the assembled system bearing host-guest (dash line).

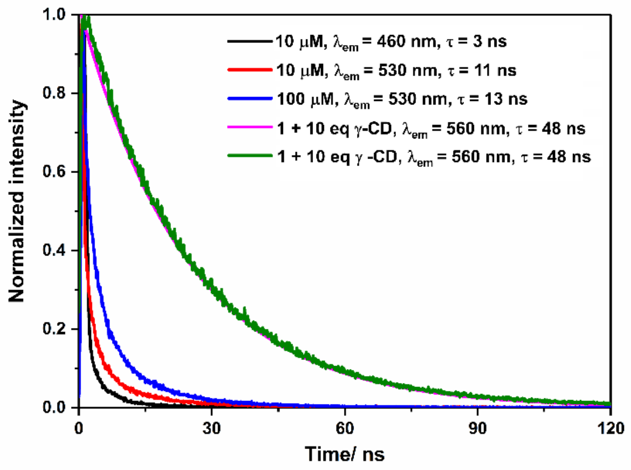

Supplementary Figure 34. Fluorescence decay at different wavelength of compound 1 (pink line: 10 μM, green line:100 μM) before and after the addition of γ-cyclodextrins, λ_{ex} = 365 nm.

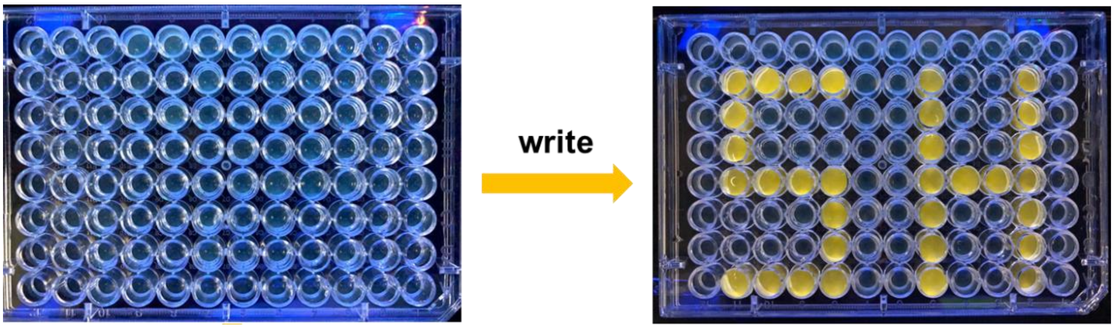

Supplementary Figure 35. Two-dimensional fluorescent photopattern in H_2O, adding 10 equiv γ-cyclodextrins (for letters “SH”) and without γ-cyclodextrins (for the other parts of the 96 well plate). Concentration: 5 μM.

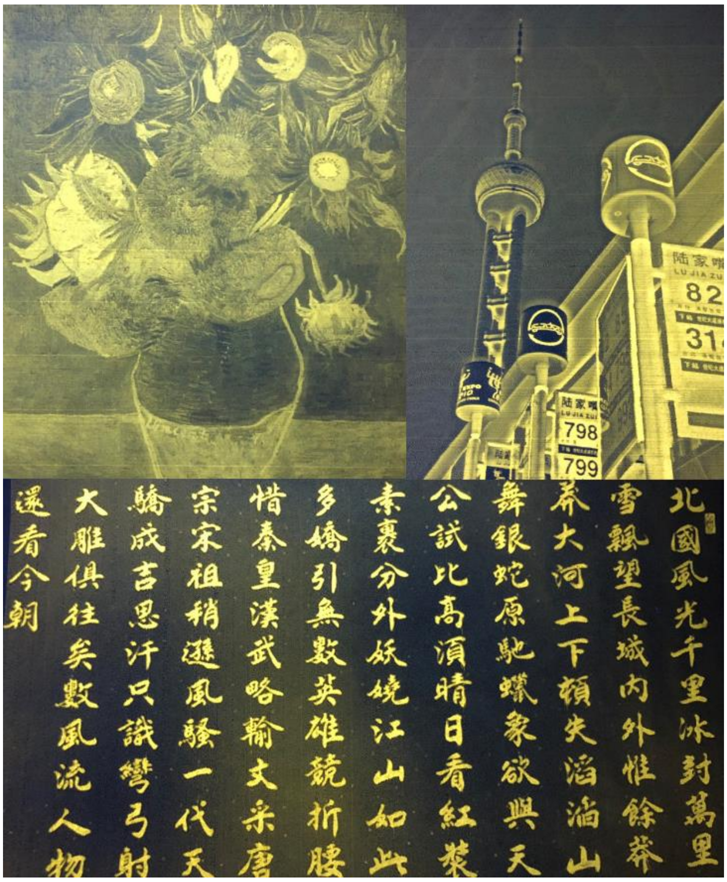

Supplementary Figure 36. Fluorescent images printed using an inkjet cartridge under UV light, in which the channel was loaded with the mixed solution of γ-cyclodextrins and compound 1.

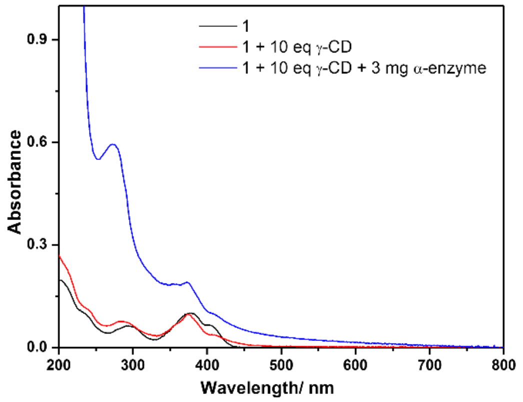

Supplementary Figure 37. Changes in the UV-Vis spectra of compound 1 (3 μM) before and after the addition of γ-CDs and enzyme.

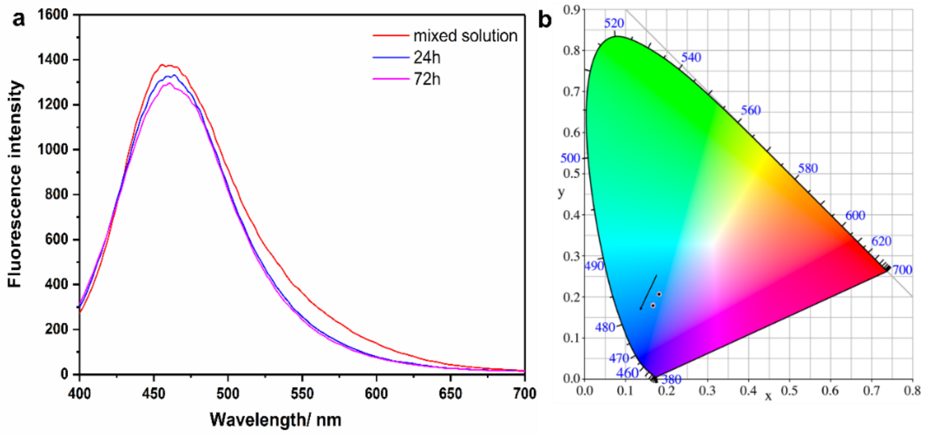

Supplementary Figure 38. (a) Changes in the fluorescence spectra of mixed solution (compound 1 3 μM, α-amylase 1 mg mL^{-1}, phosphate buffer PH = 5.2). No remarkable changes were observed after 72 hours. (b) The chromaticity coordinates (CIE) of mixed solution.

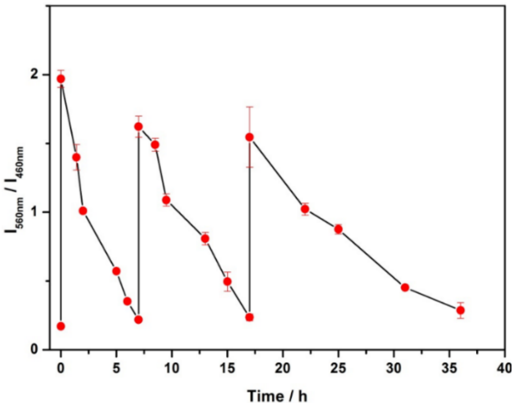

Supplementary Figure 39. Time-dependent relative fluorescence intensity (I_{560nm}/I_{460nm}) of compound 1 in H_2O (compound 1: 5 μM, α-amylase 1 mg mL^{-1}, phosphate buffer PH = 5.2). Refueling of the system was done by subsequent addition of γ-CDs (35 equiv). Data are presented as the mean ± s.d. (*n* = 3).

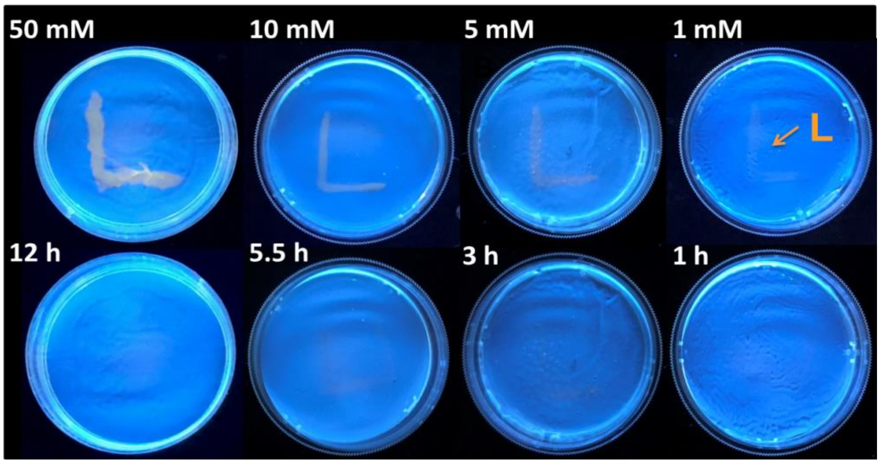

Supplementary Figure 40. The influence of ink (γ-cyclodextrins) in self-erasing gel. The lifetime of massage can be tuned by the concentration of ink. The lifetime and visibility of massage increased with the increasing concentrations of the γ-cyclodextrins.

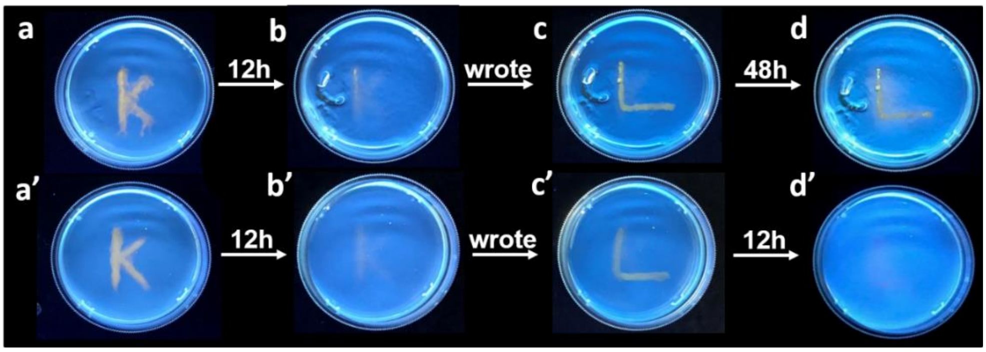

Supplementary Figure 41. The influence of α-amylase in self-erasing gel. Only once time self-erasing behavior occurred in gel a (without α-amylase), while the writing times are more than twice in gel a’ (α-amylase 1 mg mL^{-1}).

Supplementary References.

(1). Sikder, A., Das, A., & Ghosh, S. Hydrogen-bond-regulated distinct functional-group display at the inner and outer wall of vesicles. *Angew. Chem. Int. Ed.* **54**, 6755-6760 (2015).

(2). Molla, M. R. & Ghosh, S. Hydrogen-bonding-mediated vesicular assembly of functionalized naphthalene-diimide-based bolaamphiphile and guest-induced gelation in water. *Chem. Eur. J.* **18**, 9860-9869 (2012).

(3). Huang, H., Li, D. M., Wang, W., Chen, Y. C., Khan, K., Song, S. & Zheng, Y. S. Effect of carboxylic acid on micelles of a neutral amphiphilic dendro-calix[4]arene. *Org. Biomol. Chem.* **10**, 729-735 (2012).

(4). Zhang, Q. W., Li, D., Li, X., White, P. B., Mecinovic, J., Ma, X., Agren, H., Nolte, R. J. M. & Tian, H. Multicolor photoluminescence including white-light emission by a single host-guest complex. *J. Am. Chem. Soc.* **138**,13541-13550 (2016).
